# Sex-related disparities in incidence and in-hospital outcomes of Atrial fibrillation complicated by non-ST-elevation myocardial infarction from the national in-sample database (2016–2022)

**DOI:** 10.1016/j.ijcha.2025.101728

**Published:** 2025-06-25

**Authors:** Farah Yasmin, Afsana Ansari Shaik, Muhammad Sohaib Asghar, Afia Salman, Abdul Moeed, Maryam Shaharyar, Rohan Ochani, M.Chadi Alraies

**Affiliations:** a Yale School of Medicine, New Haven, CT, USA; b Mayo Clinic Rochester, MN, USA; c AdventHealth Sebring, Florida, USA; d Dow University of Health Sciences, Karachi, Pakistan; e Karachi Medical and Dental College, Karachi, Pakistan; f SUNY Upstate Medical University, NY, USA; g Detroit Medical Centre, MI, USA

**Keywords:** AF, NSTEMI, Hospital outcomes, Mortality, Cardiac catheterization

## Abstract

**Introduction:**

Atrial fibrillation (AF) is the most prevalent cardiac arrhythmia, and if complicated by NSTEMI has a significant association with a higher all-cause and cardiovascular mortality. Previous studies show that patients with AF complicated by NSTEMI have a comparatively worse prognosis than AF patients without NSTEMI.

**Methods:**

In this study, we aimed to assess the trends and gender-based disparities in the incidence and in-hospital outcomes of AF complicated by NSTEMI in the U.S. adult population, using data from the Nationwide Inpatient Sample (NIS) dataset. The NIS database was searched from 2016 to 2022 to identify patients with ICD-10 Codes. Baseline demographic characteristics along with hospital-level variables were analyzed subsequently using the odds ratio.

**Results:**

The demographic, clinical, and hospital-related characteristics of the study population were stratified by gender including 125,340 males and 88,554 females. The average age of patients was 75.14 years, with males being younger (73.58 years) compared to females (77.35 years) (p < 0.001). Overall, 10.8 % of the patients died; females had a significantly higher mortality rate (11.3 %) than males (10.5 %) (p < 0.001). The trends in age-adjusted mortality rate (AAMR) was 19996.9 per 100,000 hospitalizations in males who demonstrated higher AAMRs than females (18156.5). The mean length of hospital stay was consistently longer for the males compared to the females. The trend analysis for inflation-adjusted hospital costs demonstrated an upward trend in both males (p < 0.001) and females (p < 0.001).

**Conclusion:**

These findings underscore the importance of addressing gender-specific differences in the management of AF patients with NSTEMI to optimize patient resource allocation.

## Highlights

• **What is already known**
• AF complicated by NSTEMI has a significant association with a higher all-cause and cardiovascular mortality and patients with AF complicated by NSTEMI have a comparatively worse prognosis than AF patients without NSTEMI.
• **How do the findings change practice?**
• Our study revealed significant sex-based disparities in the incidence, clinical characteristics, resource utilization, and in-hospital outcomes of AF complicated by NSTEMI in the U.S. adult population.

## 1 Introduction

Atrial fibrillation (AF) is the most prevalent cardiac arrhythmia, with an estimated worldwide prevalence of 59 million according to the Global Burden of Disease (2019) study [1]. The national prevalence of diagnosed AF in the United States is estimated to be 10.55 million in 2019 [2]. AF is associated with increased mortality from stroke, sudden cardiac death, and cerebrovascular insult [3]. AF may present with acute myocardial infarction (MI) including non-ST-segment elevation MI (NSTEMI) [4,5]. AF complicated by NSTEMI has a significant association with a higher all-cause and cardiovascular mortality. Additionally, NSTEMI may worsen the long-term prognosis of AF [6].

Ogunbayo *et al.* demonstrated that patients with AF complicated by NSTEMI have a comparatively worse prognosis than AF patients without NSTEMI [5]. Early cardiac catheterization improves clinical outcomes in NSTEMI patients [7]. However, the existing evidence suggests age, gender, and race-based disparities in NSTEMI management including in-hospital outcomes and the incidence of complications [8]. Nonetheless, there is a paucity of research and data regarding the sex-related disparities in the incidence and clinical outcomes of AF complicated by NSTEMI. In this study, we aimed to assess the trends and gender-based disparities in the incidence and in-hospital outcomes of AF complicated by NSTEMI in the U.S. adult population, using data from the Nationwide Inpatient Sample (NIS) dataset.

## 2 Methods

The NIS database was searched from 2016 to 2022 to identify patients with AF (ICD-10 Codes: I480, I481, I482 & I4891) followed by NSTEMI type 1 (ICD-10 Codes: I214 & I219). Patients without NSTEMI (MI type 2) and those with missing data were excluded (Supplementary Fig. 1).

Hospital discharge weights were applied to estimate the sample as a total representation of the national-level in-hospital burden of the disease. The final patient population comprised more than 1.06 million weighted observations, representing 213,894 AF patients with NSTEMI. The descriptive statistics – frequencies, percents, means, and standard deviation (SD) were reported. Baseline social and demographic characteristics along with hospital-level variables were analyzed subsequently using the odds ratio. Multivariate regression analysis was used to estimate primary endpoints for in-hospital outcomes including in-patient mortality and discharge to intermediate care/skilled nursing facility which were adjusted with baseline characteristics of the study participants. Key variables adjusted included demographic characteristics (including age, gender, and race), Charlson comorbidity index, hospital-level factors (including teaching status, hospital bed-size, urban–rural location, and ownership), and socioeconomic status (including median household income per ZIP code in quartiles along with insurance status).

Adjusted odds ratios (aOR) with 95 % confidence intervals (CI) were reported. Trend analysis with the Cochran-Armitage test was used for assessing the primary outcomes with interaction analysis. The Jonckheere-Terpstra test for ordered differences were utilized to assess hospital resource utilization for all secondary outcomes. Secondary outcomes included length of hospital stay and inflation-adjusted costs. The trends were assessed using weighted data by applying discharge weights and hospital strata according to the datasets by Healthcare Cost and Utilization Project (HCUP). For cost-based analysis, total charges were converted to cost-to-charge ratio and further adjusted for inflation according to the U.S. Department of Labor inflation index for Medical Care Consumer Price Index (CPI) to adjust for inflation in 2022.

## 3 Results

Demographic, clinical, and hospital-related characteristics of the study population were stratified by gender, comprising 125,340 males and 88,554 females (Supplementary Table 1). The overall average age was 75.14 years, with males being significantly younger than females (73.58 *versus* 77.35 years; *p* < 0.001). The overall in-hospital mortality rate was 10.8 %, with females experiencing a significantly higher mortality rate compared to males (11.3 % *versus* 10.5 %; *p* < 0.001). Emergency department admissions were more common among females than males (77.5 % *versus* 74.0 %; *p* < 0.001), whereas males had significantly higher rates of elective admissions (*p* < 0.001) and longer lengths of hospital stay (*p* < 0.001). Total hospital charges and adjusted hospital costs were also significantly higher in males compared to females (*p* < 0.001 for both). In terms of discharge disposition, males were more frequently discharged to routine care (40.3 % *versus* 31.9 %; *p* < 0.001), while a greater proportion of females were discharged to intermediate or skilled nursing facilities (29.4 % *versus* 22.1 %; *p* < 0.001). Annual trends in hospital admissions from 2016 to 2022 showed a consistently higher number of male admissions compared to females each year (*p* < 0.001), although the total number of admissions varied slightly over time.

**Fig. 1:**
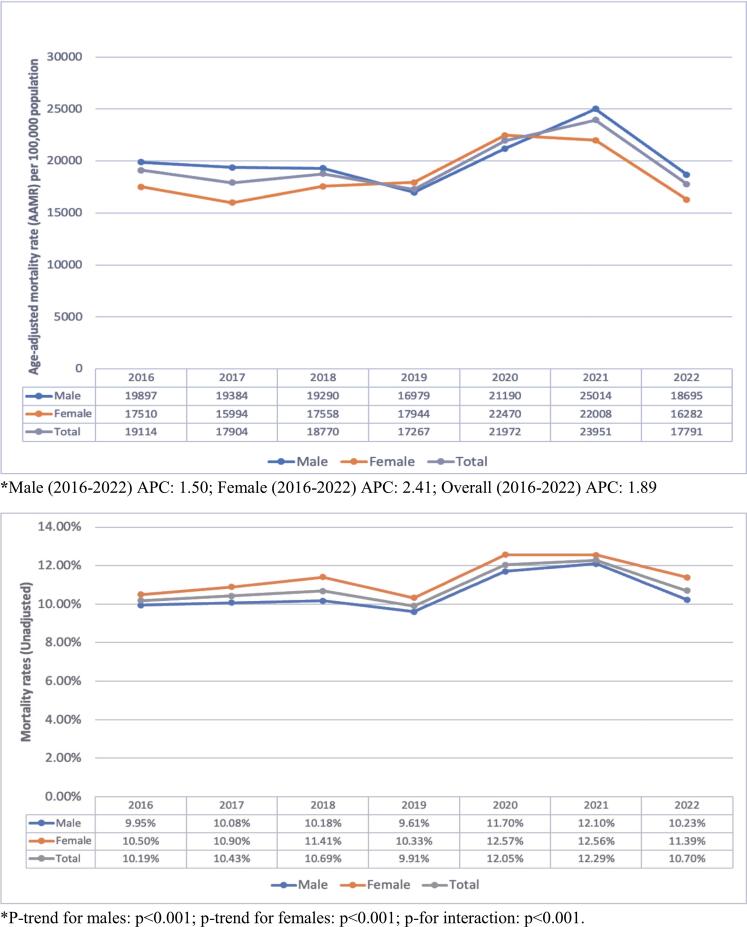
Sex-stratified trends in (A) Age-adjusted mortality rate and (B) In-hospital crude mortality for AFib patients with NSTEMI.

The prevalence of Elixhauser comorbidities, past medical history, and in-hospital events among male and female AF patients with NSTEMI are represented in Supplementary Table 2. The trends in age-adjusted mortality rate (AAMR) per 100,000 population from 2016 to 2022 demonstrated significant fluctuations in the overall population, with a sharp increase in AAMR in 2021, likely related to the COVID-19 pandemic (Fig. 1a). The overall AAMR was 19996.9 per 100,000 hospitalizations in males who demonstrated higher AAMRs than females (18156.5). The trend analysis for unadjusted in-hospital mortality rates suggested an overall upward trend, notably between 2019–2021 (Fig. 1b). The Cochran-Armitage test indicated a significantly higher in-hospital mortality rate among females compared to males (p < 0.001). Sex-stratified trends in the mean length of hospital stay during 2016–2022 is demonstrated in Supplementary Fig. 2. The Jonckheere-Terpstra test revealed a statistically significant overall downward trend over the years for males (p < 0.001) and females (p < 0.001), exhibiting similar patterns of fluctuation. However, the mean length of hospital stay was consistently longer for males compared to females. The trend analysis for inflation-adjusted hospital costs stratified by gender from 2016 to 2022 demonstrated an upward trend in both males (p < 0.001) and females (p < 0.001) according to the Jonckheere-Terpstra test (Supplementary Fig. 2).

The mean hospital costs remained higher for males. The overall revascularization trends during 2016–2022 suggested an increasing trend in the PCI and CABG procedures for both males and females (p < 0.001) (Supplementary Fig. 3). The rates of both PCI and CABG procedures were consistently higher for males than for females throughout the study period (p < 0.001), from 2016 (17.36 % *versus* 12.16 % for PCI; 10.66 % *versus* 5.02 % for CABG) to 2022 (21.45 % *versus* 17.18 % for PCI; 13.86 % *versus* 6.59 % for CABG).

The findings from the multivariate regression analysis examining sex-related disparities in resource utilization and in-hospital mortality are presented in Table 1. After adjustment for potential confounders, female sex was associated with a significantly increased risk of in-hospital mortality compared to males, with an adjusted odds ratio (aOR) of 1.030 (95 % CI: 1.001–1.059; *p* = 0.043). Additionally, females incurred significantly lower inflation-adjusted hospital costs, with a mean difference of –$2,987 (95 % CI: –3,275 to –2,699; *p* < 0.001). Females also had higher odds of being discharged to a skilled nursing facility compared to males (aOR 1.332; 95 % CI: 1.304–1.360; *p* < 0.001). Supplementary Fig. 4 highlights the key predictors of in-hospital mortality among atrial fibrillation (AF) patients with non-ST elevation myocardial infarction (NSTEMI), identifying cardiac arrest, acute bowel ischemia, hemodialysis, and the need for invasive ventilation as having the highest aORs.

**Table 1:** Sex-related Disparities for Resource Utilization and In-Hospital Mortality on Multiple linear/Multivariate Regression among AFib patients with NSTEMI.

**Study Outcomes**	**Univariate, OR/regression coefficient**	**95 % confidence interval**	P-value	**Multivariate, aOR/regression coefficient**	**95 % confidence interval**	P-value
**In-hospital mortality**						
**Female**	1.085	1.056–1.116	<0.001	1.030	1.001–1.059	0.043
**Male**	Reference	−	−	Reference	−	−
**Hospital LOS**						
**Female**	−0.409	−0.472–-0.346	<0.001	−0.059	−0.121 –+0.004	0.065
**Male**	Reference	−	−	Reference	−	−
**Inflation-adjusted hospital cost**						
**Female**	−5721.01	−6014.1–-5427.9	<0.001	−2987.52	−3275.1 –-2699.9	<0.001
**Male**	Reference	−	−	Reference	−	−
**Discharge to SNF**						
**Female**	1.518	1.488–1.549	<0.001	1.332	1.304–1.360	<0.001
**Male**	Reference	−	−	Reference	−	−

*Adjusted for age, Charlson index, ED admission, weekend admission, elective admission, race, median income per ZIP quartile, insurance status, hospital region, teaching location, ownership and bedsize. SNF: Skilled nursing facility; OR: Odds ratio; LOS: Length of stay.

## 4 Discussion

Our study, utilizing the NIS database, investigated sex-related differences in AF patients with NSTEMI using a large national cohort. Females were older, had higher in-hospital mortality, and were more frequently admitted through the emergency department compared to males, who had longer hospital stays, higher costs, and were more often discharged to routine care. Females had significantly lower hospital costs and higher odds of being discharged to skilled nursing facilities. Mortality rates, hospital stays, and costs showed significant trends over time, with males consistently exhibiting higher revascularization rates (PCI and CABG). Multivariate analysis confirmed higher in-hospital mortality risk and discharge to skilled care among females, despite lower healthcare costs, underscoring persistent sex disparities in clinical outcomes and resource utilization.

While the study acknowledged the potential influence of the COVID-19 pandemic on mortality trends, further investigation is needed to understand the specific mechanisms behind this association. A significant increase in mortality was observed during the pandemic, particularly in 2020 and 2021. Among the study population, 4,734 patients (2.2 %) were diagnosed with COVID-19, which was associated with a 2.76-fold increase in the odds of mortality (95 % CI: 2.54–2.99). Interestingly, male patients had longer hospital stays than females, which contrasts with prior literature that often reports higher healthcare resource utilization among females [9]. In general, individuals aged 45 to 64 exhibit the greatest disparities in healthcare spending, often due to menopausal symptoms and chronic illnesses that predominantly affect women in this age range, such as osteoporosis, breast cancer, and cardiovascular disease which rises sharply with the onset of menopause. However, in this study of patients with AF and NSTEMI, males consistently showed greater resource use regardless of age, as evidenced by longer hospital stays and higher hospitalization costs. Age did not appear to be a determining factor in these differences, as male patients were notably younger than female patients (73.6 vs. 77.4 years).

The study found that although males had a higher age-adjusted mortality rate (AAMR), females exhibited a higher crude in-hospital mortality rate. This seemingly contradictory finding is largely attributable to the age difference between the sexes in the study cohort, with females being older than males (77 vs. 74 years). After adjusting for age, certain years within the study period—particularly 2019 and 2020—still showed higher age-adjusted mortality rates in females, while males maintained higher overall mortality in the years before and after. The unadjusted in-hospital mortality rate was 11.3 % in females compared to 10.5 % in males (OR: 1.085 [1.056–1.116]). When adjusted for age and other baseline characteristics, the multivariate analysis yielded an adjusted odds ratio (aOR) of 1.030 [1.001–1.059], with borderline statistical significance (p = 0.043). Adjustment for age alone resulted in an OR of 1.003 [0.975–1.032], indicating no significant difference (p = 0.836), while age itself was a strong predictor of mortality, with an OR of 1.022 [1.021–1.024], (p < 0.001).

Notwithstanding, there were several limitations in our analysis. Firstly, the NIS relies on administrative data and ICD codes, which do not include granular clinical information such as laboratory values, imaging results, medications, or timing of clinical events. Second, the data depend on accurate coding by hospitals; misclassification or inconsistencies in ICD coding can lead to biased or inaccurate results. Third, the NIS is a discharge-level dataset, meaning it does not allow for patient-level tracking across multiple admissions or long-term follow-up. Last. The NIS only captures data from inpatient hospitalizations, so findings may not be generalizable to patients treated in outpatient or emergency department settings without admission.

## 5 Conclusion

These findings underscore the importance of recognizing and addressing gender-specific differences in the management of AF patients with NSTEMI to optimize patient outcomes and resource allocation.

## Authorship statement

This author takes responsibility for all aspects of the reliability and freedom from bias of the data presented and their discussed interpretation.

## Ethical approval

Ethical approval not required to conduct the study and IRB waiver was obtained from AdventHealth Research Institute IRBnet#2291268.

## Informed consent to participate

As the HCUP NIS database lacks patient and hospital identifiers, informed consent was not required for this study and was waived by human subject determination on behalf of IRB committee.

## Data availability statement

HCUP is an online system that makes the NIS resources available to public health professionals and the public. The system provides access to a wide array of public health information on hospitalized patients. HCUP furthers mission of health promotion and disease for healthcare cost and resource utilization by speeding up and simplifying access to public health information. NIS analysis is valuable in public health research, decision-making, priority setting, program evaluation, and resource allocation. The HCUP project is publicly available at https://hcup-us.ahrq.gov/databases.jsp/). All the datasets generated from the above resources can be made available on a reasonable request from the corresponding author. No datasets can be made publicly available on a data repository platform without taking the adequate permission levels from the HCUP.

## CRediT authorship contribution statement

**Farah Yasmin:** Writing – review & editing, Project administration, Conceptualization. **Afsana Ansari Shaik:** Writing – original draft, Formal analysis, Data curation. **Muhammad Sohaib Asghar:** Methodology, Formal analysis, Data curation. **Afia Salman:** Writing – original draft, Resources, Investigation. **Abdul Moeed:** Writing – original draft, Validation, Software. **Maryam Shaharyar:** Writing – review & editing, Visualization, Investigation. **Rohan Ochani:** Writing – review & editing, Validation, Project administration. **M.Chadi Alraies:** Writing – review & editing, Software, Project administration.

## Funding

No funding was used to conduct the study.

## Declaration of competing interest

The authors declare that they have no known competing financial interests or personal relationships that could have appeared to influence the work reported in this paper.
