# Supplementary material for: Sex-related disparities in incidence and in-hospital outcomes of Atrial fibrillation complicated by non-ST-elevation myocardial infarction from the national in-sample database (2016–2022)
Source: Int J Cardiol Heart Vasc. 2025 Jun 25;59:101728. doi: 10.1016/j.ijcha.2025.101728 (PMC12859438; doi:10.1016/j.ijcha.2025.101728)
Supplement: Supplementary Data 1 [file mmc1.docx]

**Sex-related Disparities in Incidence and In-Hospital Outcomes of Atrial Fibrillation complicated by Non-ST-Elevation Myocardial Infarction.**

Supplementary Figure 1: NIS database screening.

**Identification of patient records from the NIS database (2016-2022)**

- ICD-codes applied to capture patients with atrial fibrillation (n=5,593,184).

**42,607,192 unweighted observations removed.

**Identification**

Records identified from

NIS Database 2016-2022

(n = 48,200,376) *

**Equivalent to Weighted data of 241,001,774 (>241 million)*

Records excluded.

- Patients without NSTEMI: n=5,366,485

**26,832,413 weighted observations removed.

**Screening**

Records screened

(n = 5,593,184) *

**Equivalent to Weighted data of 27,965,908 (>27.9 million)*

Patient records not retrievable (n=0)

Number of patients retrieved

(n = 226,699)

Records excluded for missing data:

(n = 12,805)

** 64,025 weighted observations removed.

Reports assessed for eligibility

(n = 226,699) *

**Equivalent to Weighted data of 1,133,495 (>1.13 million)*

**Included**

Patient’s records included in the study

(n = 213,894)

**Equivalent to Weighted data of 1,069,470 (>1.06 million)*

*Hospital discharge weights obtained from NIS – HCUP defined stratum and primary sampling units (PSU) of hospital universe to provide national level estimates.

**Records removed due to exclusion criteria.

| **Supplementary Table. 1 Baseline Characteristics of the Study Population.** | | | | |
| --- | --- | --- | --- | --- |
| Variables | **Total (n=213,894)** | **Male (n=125,340)** | **Female (n=88,554)** | **p-value** |
| **Age** mean (SD) | 75.14 (11.03) | 73.58 (11.15) | 77.35 (10.48) | <0.001 |
| **Mortality** n (%) | 23071 (10.8%) | 13107 (10.5%) | 9964 (11.3%) | <0.001 |
| **Elective admission** n (%) | 12346 (5.8%) | 7517 (6%) | 4829 (5.5%) | <0.001 |
| **Weekend admission** n (%) | 54316 (25.4%) | 31369 (25%) | 22947 (25.9%) | <0.001 |
| **ED admission** n (%) | 161317 (75.4%) | 92697 (74%) | 68620 (77.5%) | <0.001 |
| **LOS** mean (SD) | 7.11 (7.30) | 7.28 (7.50) | 6.87 (7.00) | <0.001 |
| **Total hospital charges in $** mean (SD) | 120306.7 (157913.1) | 130288.8 (168315.4) | 106177.9 (140687.2) | <0.001 |
| **Total cost (adjusted for inflation)**  **in $** mean (SD) | 29421.9 (34185.3) | 31790.4 (36174.0) | 26069.4 (30844.9) | <0.001 |
| **Disposition** n (%) | | | | <0.001 |
| Routine (alive) | 78716 (36.8%) | 50505 (40.3%) | 28211 (31.9%) |  |
| Short-term facility | 13924 (6.5%) | 8672 (6.9%) | 5252 (5.9%) |  |
| Intermediate or SNF | 53725 (25.1%) | 27657 (22.1%) | 26068 (29.4%) |  |
| Home health | 42496 (19.9%) | 23955 (19.1%) | 18541 (20.9%) |  |
| AMA | 1962 (0.9%) | 1444 (1.2%) | 518 (0.6%) |  |
| Died | 23071 (10.8%) | 13107 (10.5%) | 9964 (11.3%) |  |
| **Discharge quarter** n (%) | | | | 0.694 |
| January-March | 57537 (26.9%) | 33791 (27%) | 23746 (26.8%) |  |
| April-June | 52733 (24.7%) | 30956 (24.7%) | 21777 (24.6%) |  |
| July-September | 51272 (24%) | 29951 (23.9%) | 21321 (24.1%) |  |
| October-December | 52352 (24.5%) | 30642 (24.4%) | 21710 (24.5%) |  |
| **Health Insurance** n (%) | | | | <0.001 |
| Medicare | 170099 (79.5%) | 94569 (75.4%) | 75530 (85.3%) |  |
| Medicaid | 10318 (4.8%) | 6517 (5.2%) | 3801 (4.3%) |  |
| Private including HMO | 24997 (11.7%) | 17544 (14%) | 7453 (8.4%) |  |
| Self-pay | 3157 (1.5%) | 2240 (1.8%) | 917 (1%) |  |
| No charge or others | 5323 (2.5%) | 4470 (3.6%) | 853 (1%) |  |
| **NCHS urbanization scheme** n (%) | | | | <0.001 |
| Large metropolitan (>1 million population) | 105846 (49.5%) | 61563 (49.1%) | 44283 (50%) |  |
| Medium-small metropolitan (< 1 million population) | 64745 (30.3%) | 38348 (30.6%) | 26397 (29.8%) |  |
| Non-metropolitan | 43303 (20.2%) | 25429 (20.3%) | 17874 (20.2%) |  |
| **Race** n (%) | | | | <0.001 |
| White | 169019 (79%) | 100168 (79.9%) | 68851 (77.8%) |  |
| Black | 18146 (8.5%) | 9364 (7.5%) | 8782 (9.9%) |  |
| Hispanic | 14955 (7%) | 8829 (7%) | 6126 (6.9%) |  |
| Others | 11774 (5.5%) | 6979 (5.6%) | 4795 (5.4%) |  |
| **Median household income quartiles per area zip code** n (%) | | | | <0.001 |
| $1 – 55999 $ (0–25^th^ percentile) | 63463 (29.7%) | 36101 (28.8%) | 27362 (30.9%) |  |
| $ 56000 – $ 70999 (26–50^th^ percentile) | 59316 (27.7%) | 34473 (27.5%) | 24843 (28.1%) |  |
| $ 71000 - $ 93999 (51–75^th^ percentile) | 50391 (23.6%) | 30062 (24%) | 20329 (23%) |  |
| $ 94000 – or more (76–100^th^ percentile) | 40724 (19%) | 24704 (19.7%) | 16020 (18.1%) |  |
| **Hospital bed size** n (%) | | | | <0.001 |
| Small | 42495 (19.9%) | 23844 (19%) | 18651 (21.1%) |  |
| Medium | 65196 (30.5%) | 37861 (30.2%) | 27335 (30.9%) |  |
| Large | 106203 (49.7%) | 63635 (50.8%) | 42568 (48.1%) |  |
| **Hospital location and teaching services** n (%) | | | | <0.001 |
| Rural | 18990 (8.9%) | 10169 (8.1%) | 8821 (10%) |  |
| Urban, non-teaching | 45987 (21.5%) | 26409 (21.1%) | 19578 (22.1%) |  |
| Urban, teaching | 148917 (69.6%) | 88762 (70.8%) | 60155 (67.9%) |  |
| **Hospital region** n (%) | | | | <0.001 |
| Northeast | 39150 (18.3%) | 22638 (18.1%) | 16512 (18.6%) |  |
| Midwest | 48581 (22.7%) | 28361 (22.6%) | 20220 (22.8%) |  |
| South | 85720 (40.1%) | 50150 (40%) | 35570 (40.2%) |  |
| West | 40443 (18.9%) | 24191 (19.3%) | 16252 (18.4%) |  |
| **Transfer into the hospital** n (%) | | | | <0.001 |
| Not transferred | 166730 (78.4%) | 96516 (77.5%) | 70214 (79.8%) |  |
| Transfer in from another hospital | 35110 (16.5%) | 22384 (18%) | 12726 (14.5%) |  |
| Transfer from SNF/other facility | 10717 (5%) | 5656 (4.5%) | 5061 (5.8%) |  |
| **Transfer out of the hospital** n (%) | | | | <0.001 |
| Not transferred | 146245 (68.4%) | 89011 (71%) | 57234 (64.6%) |  |
| Transfer to another acute care hospital | 13924 (6.5%) | 8672 (6.9%) | 5252 (5.9%) |  |
| Transfer to SNF/other facility | 53725 (25.1%) | 27657 (22.1%) | 26068 (29.4%) |  |
| **DRG: Disease Severity Subclass** n (%) | | | | <0.001 |
| Minor-moderate | 54475 (25.5%) | 33150 (26.4%) | 21325 (24.1%) |  |
| Severe | 74271 (34.7%) | 43238 (34.5%) | 31033 (35%) |  |
| Extreme | 85146 (39.8%) | 48950 (39.1%) | 36196 (40.9%) |  |
| **DRG: Risk of Mortality Subclass** n (%) | | | | <0.001 |
| Minor-moderate | 39654 (18.5%) | 25535 (20.4%) | 14119 (15.9%) |  |
| Severe | 83373 (39%) | 47281 (37.7%) | 36092 (40.8%) |  |
| Extreme | 90865 (42.5%) | 52522 (41.9%) | 38343 (43.3%) |  |
| **Hospital ownership** n (%) | | | | 0.009 |
| Government, non-federal | 17629 (8.2%) | 10377 (8.3%) | 7252 (8.2%) |  |
| Private, non-profit | 160341 (75%) | 94173 (75.1%) | 66168 (74.7%) |  |
| Private, investor-own | 35924 (16.8%) | 20790 (16.6%) | 15134 (17.1%) |  |
| **Year of index admission** n (%) | | | | <0.001 |
| 2016 | 36630 (17.1%) | 20720 (16.5%) | 15910 (18%) |  |
| 2017 | 39805 (18.6%) | 22779 (18.2%) | 17026 (19.2%) |  |
| 2018 | 32109 (15%) | 18930 (15.1%) | 13179 (14.9%) |  |
| 2019 | 30481 (14.3%) | 18036 (14.4%) | 12445 (14.1%) |  |
| 2020 | 24192 (11.3%) | 14547 (11.6%) | 9645 (10.9%) |  |
| 2021 | 25249 (11.8%) | 15186 (12.1%) | 10063 (11.4%) |  |
| 2022 | 25428 (11.9%) | 15142 (12.1%) | 10286 (11.6%) |  |
| ED: emergency; HMO: health maintenance organization; DRG: diagnosis related groups; LOS: length of stay; SNF: skilled nursing facility; AMA: against medical device; NCHS: National Center for Health Statistics.  **Median household income quartiles per area zip code quartiles for each year:**   \| Year \| 1^st^ quartile (0–25^th^ percentile) \| 2^nd^ quartile (26–50^th^ percentile) \| 3^rd^ quartile (51–75^th^ percentile) \| 4^th^ quartile (75–100^th^ percentile) \| \| --- \| --- \| --- \| --- \| --- \| \| 2016 \| 1 - 42,999 \| 43,000 - 53,999 \| 54,000 - 70,999 \| 71,000+ \| \| 2017 \| 1 - 43,999 \| 44,000 - 55,999 \| 56,000 - 73,999 \| 74,000+ \| \| 2018 \| 1 - 45,999 \| 46,000 - 58,999 \| 59,000 - 78,999 \| 79,000+ \| \| 2019 \| 1 - 47,999 \| 48,000 - 60,999 \| 61,000 - 81,999 \| 82,000+ \| \| 2020 \| 1 - 49,999 \| 50,000 - 64,999 \| 65,000 - 85,999 \| 86,000+ \| \| 2021 \| 1 - 51,999 \| 52,000 - 65,999 \| 66,000 - 87,999 \| 88,000+ \| \| 2022 \| 1 - 55,999 \| 56,000 - 70,999 \| 71,000 - 93,999 \| 94,000+ \| | | | | |

| **Supplementary Table. 2 Gender Disparities in Baseline Comorbidities and In-hospital Events of the Study Population.** | | | | |
| --- | --- | --- | --- | --- |
| **Variables** | **Total (n=213,894)** | **Male (n=125,340)** | **Female (n=88,554)** | **p-value** |
| **Elixhauser Comorbidity Index** | | | | |
| **HIV** | 433 (0.2%) | 356 (0.3%) | 77 (0.1%) | <0.001 |
| **Alcohol abuse** | 7004 (3.3%) | 5982 (4.8%) | 1022 (1.2%) | <0.001 |
| **Autoimmune conditions** | 7566 (3.5%) | 2888 (2.3%) | 4678 (5.3%) | <0.001 |
| **Coagulopathy** | 27855 (13%) | 18329 (14.6%) | 9526 (10.8%) | <0.001 |
| **Depression** | 21249 (9.9%) | 9856 (7.9%) | 11393 (12.9%) | <0.001 |
| **Diabetes (Uncomplicated)** | 36620 (17.1%) | 21677 (17.3%) | 14943 (16.9%) | 0.011 |
| **Complicated Diabetes** | 62658 (29.3%) | 38507 (30.7%) | 24151 (27.3%) | <0.001 |
| **Substance abuse** | 4752 (2.2%) | 3442 (2.7%) | 1310 (1.5%) | <0.001 |
| **Hypertension (Uncomplicated)** | 60338 (28.2%) | 34348 (27.4%) | 25990 (29.3%) | <0.001 |
| **Complicated Hypertension** | 111929 (52.3%) | 65302 (52.1%) | 46627 (52.7%) | 0.012 |
| **Mild liver disease** | 9134 (4.3%) | 6015 (4.8%) | 3119 (3.5%) | <0.001 |
| **Liver disease, moderate to severe** | 1999 (0.9%) | 1339 (1.1%) | 660 (0.7%) | <0.001 |
| **Obesity** | 40911 (19.1%) | 24021 (19.2%) | 16890 (19.1%) | 0.596 |
| **Peripheral artery disease** | 32718 (15.3%) | 20050 (16%) | 12668 (14.3%) | <0.001 |
| **Psychoses** | 4349 (2%) | 2400 (1.9%) | 1949 (2.2%) | <0.001 |
| **Pulmonary circulation disease** | 25411 (11.9%) | 12764 (10.2%) | 12647 (14.3%) | <0.001 |
| **Renal disease (moderate)** | 54755 (25.6%) | 33533 (26.8%) | 21222 (24%) | <0.001 |
| **Renal disease (severe)** | 28335 (13.2%) | 17029 (13.6%) | 11306 (12.8%) | <0.001 |
| **Weight loss** | 17889 (8.4%) | 9669 (7.7%) | 8220 (9.3%) | <0.001 |
| **Past Medical History** | | | | |
| **Prior MI** | 36044 (16.9%) | 23271 (18.6%) | 12773 (14.4%) | <0.001 |
| **Prior PCI** | 36965 (17.3%) | 24511 (19.6%) | 12454 (14.1%) | <0.001 |
| **Prior CABG** | 30176 (14.1%) | 21755 (17.4%) | 8421 (9.5%) | <0.001 |
| **Prior pacemaker** | 15333 (7.2%) | 8630 (6.9%) | 6703 (7.6%) | <0.001 |
| **Prior valve replacement** | 7659 (3.6%) | 4601 (3.7%) | 3058 (3.5%) | 0.008 |
| **Prior ICD** | 22837 (10.7%) | 14395 (11.5%) | 8442 (9.5%) | <0.001 |
| **Prior CVA (including sequalae)** | 30681 (14.3%) | 16801 (13.4%) | 13880 (15.7%) | <0.001 |
| **Prior peripheral angioplasty** | 1308 (0.6%) | 803 (0.6%) | 505 (0.6%) | 0.040 |
| **Cardiovascular** | | | | |
| **Coronary artery disease** | 155244 (72.6%) | 97761 (78%) | 57483 (64.9%) | <0.001 |
| **Heart Failure** | 133958 (62.6%) | 77599 (61.9%) | 56359 (63.6%) |  |
| **Acute exacerbation of heart failure** | 73055 (34.2%) | 39994 (31.9%) | 33061 (37.3%) | <0.001 |
| **Systolic heart failure** | 74629 (34.9%) | 48117 (38.4%) | 26512 (29.9%) | <0.001 |
| **Diastolic heart failure** | 60013 (28.1%) | 31067 (24.8%) | 28946 (32.7%) | <0.001 |
| **Valvular heart disease** | 55273 (25.8%) | 30789 (24.6%) | 24484 (27.6%) | <0.001 |
| **Mitral stenosis** | 840 (0.4%) | 239 (0.2%) | 601 (0.7%) | <0.001 |
| **Mitral valve disease** | 15668 (7.3%) | 8221 (6.6%) | 7447 (8.4%) | <0.001 |
| **Carotid stenosis** | 7984 (3.7%) | 4766 (3.8%) | 3218 (3.6%) | 0.043 |
| **Pericarditis/Pericardial effusion** | 4443 (2.1%) | 2590 (2.1%) | 1853 (2.1%) | 0.676 |
| **Pericardial Tamponade** | 457 (0.2%) | 265 (0.2%) | 192 (0.2%) | 0.790 |
| **Rheumatic heart disease** | 17845 (8.3%) | 8770 (7%) | 9075 (10.2%) | <0.001 |
| **Takotsubo cardiomyopathy** | 1996 (0.9%) | 382 (0.3%) | 1614 (1.8%) | <0.001 |
| **Peripheral atherosclerosis** | 9138 (4.3%) | 5138 (4.1%) | 4000 (4.5%) | <0.001 |
| **Infective endocarditis** | 1617 (0.8%) | 995 (0.8%) | 622 (0.7%) | 0.016 |
| **Cardiac rhythm abnormalities** | | | | |
| **SVT** | 9920 (4.6%) | 5663 (4.5%) | 4257 (4.8%) | 0.002 |
| **VT** | 16424 (7.7%) | 11561 (9.2%) | 4863 (5.5%) | <0.001 |
| **Sick Sinus Syndrome** | 7889 (3.7%) | 4168 (3.3%) | 3721 (4.2%) | <0.001 |
| **AV block** | 8938 (4.2%) | 5868 (4.7%) | 3070 (3.5%) | <0.001 |
| **Pulmonary** | | | | |
| **Respiratory failure (acute and chronic)** | 68035 (31.8%) | 38738 (30.9%) | 29297 (33.1%) | <0.001 |
| **Acute respiratory failure** | 62079 (29%) | 35795 (28.6%) | 26284 (29.7%) | <0.001 |
| **Respiratory complications** | 4816 (2.3%) | 3282 (2.6%) | 1534 (1.7%) |  |
| **Respiratory tract bleeding** | 3514 (1.6%) | 2275 (1.8%) | 1239 (1.4%) | <0.001 |
| **Smoking history** | 83553 (39.1%) | 56878 (45.4%) | 26675 (30.1%) | <0.001 |
| **Pneumonia** | 45218 (21.1%) | 26699 (21.3%) | 18519 (20.9%) | 0.030 |
| **Aspiration pneumonitis/pneumonia** | 10944 (5.1%) | 6775 (5.4%) | 4169 (4.7%) | <0.001 |
| **BiPAP dependence** | 1593 (0.7%) | 998 (0.8%) | 595 (0.7%) | 0.001 |
| **Oxygen dependence** | 9335 (4.4%) | 4675 (3.7%) | 4660 (5.3%) | <0.001 |
| **Pulmonary hypertension** | 25525 (11.9%) | 12829 (10.2%) | 12696 (14.3%) | <0.001 |
| **OSA** | 23152 (10.8%) | 16312 (13%) | 6840 (7.7%) | <0.001 |
| **Pneumothorax** | 2614 (1.2%) | 1827 (1.5%) | 787 (0.9%) | <0.001 |
| **Chronic pulmonary diseases** | 63987 (29.9%) | 36193 (28.9%) | 27794 (31.4%) | <0.001 |
| **COPD** | 56750 (26.5%) | 32997 (26.3%) | 23753 (26.8%) | 0.010 |
| **Acute exacerbation of COPD** | 19673 (9.2%) | 11200 (8.9%) | 8473 (9.6%) | <0.001 |
| **Acute asthma** | 947 (0.4%) | 360 (0.3%) | 587 (0.7%) | <0.001 |
| **Acute bronchitis** | 2343 (1.1%) | 1279 (1%) | 1064 (1.2%) | <0.001 |
| **COVID-19** | 4734 (2.2%) | 2844 (2.3%) | 1890 (2.1%) | 0.037 |
| **Neurological** | | | | |
| **Cerebrovascular disease** | 24844 (11.6%) | 13683 (10.9%) | 11161 (12.6%) | <0.001 |
| **Neurological disorders affecting movements** | 6542 (3.1%) | 3629 (2.9%) | 2913 (3.3%) | <0.001 |
| **Other neurological disorders** | 26555 (12.4%) | 14774 (11.8%) | 11781 (13.3%) | <0.001 |
| **Seizures and epilepsy** | 7021 (3.3%) | 3961 (3.2%) | 3060 (3.5%) | <0.001 |
| **Status epilepticus** | 379 (0.2%) | 199 (0.2%) | 180 (0.2%) | 0.016 |
| **Paralysis** | 11703 (5.5%) | 6275 (5%) | 5428 (6.1%) | <0.001 |
| **Migraine** | 1152 (0.5%) | 405 (0.3%) | 747 (0.8%) | <0.001 |
| **TIA** | 1135 (0.5%) | 557 (0.4%) | 578 (0.7%) | <0.001 |
| **Stroke** | 8705 (4.1%) | 4357 (3.5%) | 4348 (4.9%) | <0.001 |
| **Dementia** | 23984 (11.2%) | 11321 (9%) | 12663 (14.3%) | <0.001 |
| **Gastrointestinal** | | | | |
| **UGI Bleeding** | 10463 (4.9%) | 6228 (5%) | 4235 (4.8%) | 0.049 |
| **LGI Bleeding** | 5951 (2.8%) | 3453 (2.8%) | 2498 (2.8%) | 0.361 |
| **Peptic ulcer with bleeding** | 3654 (1.7%) | 2234 (1.8%) | 1420 (1.6%) | 0.002 |
| **IBD** | 1270 (0.6%) | 697 (0.6%) | 573 (0.6%) | 0.007 |
| **Acute bowel ischemia** | 464 (0.2%) | 199 (0.2%) | 265 (0.3%) | <0.001 |
| **Endocrine** | | | | |
| **Diabetes** | 96813 (45.3%) | 58806 (46.9%) | 38007 (42.9%) | <0.001 |
| **Hypertension** | 167440 (78.3%) | 97071 (77.4%) | 70369 (79.5%) | <0.001 |
| **Diabetic ketoacidosis** | 2133 (1%) | 1102 (0.9%) | 1031 (1.2%) | <0.001 |
| **Hypothyroidism** | 37272 (17.4%) | 14603 (11.7%) | 22669 (25.6%) | <0.001 |
| **Other thyroid disorders (including hyperthyroidism)** | 3076 (1.4%) | 1145 (0.9%) | 1931 (2.2%) | <0.001 |
| **Dyslipidemia** | 126295 (59%) | 76495 (61%) | 49800 (56.2%) | <0.001 |
| **Major In-hospital events and procedures** | | | | |
| **Septic shock** | 14741 (6.9%) | 8521 (6.8%) | 6220 (7%) | 0.042 |
| **Cardiogenic shock** | 14284 (6.7%) | 9187 (7.3%) | 5097 (5.8%) | <0.001 |
| **Major bleeding** | 3075 (1.4%) | 1764 (1.4%) | 1311 (1.5%) | 0.162 |
| **In-hospital cardiac arrest** | 9976 (4.7%) | 6317 (5%) | 3659 (4.1%) | <0.001 |
| **Mechanical circulatory support** | 27991 (13.1%) | 20180 (16.1%) | 7811 (8.8%) | <0.001 |
| **In-hospital PRBC transfusion** | 17644 (8.2%) | 10083 (8%) | 7561 (8.5%) | <0.001 |
| **In-hospital PCI** | 37899 (17.7%) | 24493 (19.5%) | 13406 (15.1%) | <0.001 |
| **In-hospital CABG** | 21504 (10.1%) | 16192 (12.9%) | 5312 (6%) | <0.001 |
| **In-hospital pacemaker insertion** | 3430 (1.6%) | 1892 (1.5%) | 1538 (1.7%) | <0.001 |
| **Invasive mechanical ventilation** | 27834 (13%) | 17027 (13.6%) | 10807 (12.2%) | <0.001 |
| **Tracheostomy** | 21609 (10.1%) | 13106 (10.5%) | 8503 (9.6%) | <0.001 |
| **PEG tube placement** | 4690 (2.2%) | 2881 (2.3%) | 1809 (2%) | <0.001 |
| **Acute pulmonary embolism** | 3165 (1.5%) | 1773 (1.4%) | 1392 (1.6%) | 0.003 |
| **Acute VTE** | 4674 (2.2%) | 2750 (2.2%) | 1924 (2.2%) | 0.739 |
| **In-hospital catheter ablation** | 975 (0.5%) | 690 (0.6%) | 285 (0.3%) | <0.001 |
| **In-hospital defibrillator** | 1816 (0.8%) | 1398 (1.1%) | 418 (0.5%) | <0.001 |
| **Mitral valve replacement** | 706 (0.3%) | 427 (0.3%) | 279 (0.3%) | 0.309 |
| **Hematology/Oncology** | | | | |
| **Any malignancy** | 14257 (6.7%) | 9304 (7.4%) | 4953 (5.6%) | <0.001 |
| **Leukemia** | 1774 (0.8%) | 1213 (1%) | 561 (0.6%) | <0.001 |
| **Lymphoma** | 2082 (1%) | 1378 (1.1%) | 704 (0.8%) | <0.001 |
| **Metastatic cancer** | 3884 (1.8%) | 2490 (2%) | 1394 (1.6%) | <0.001 |
| **Solid organ malignancies** | 9650 (4.5%) | 6299 (5%) | 3351 (3.8%) | <0.001 |
| **Deficiency anemias** | 60851 (28.4%) | 34276 (27.3%) | 26575 (30%) | <0.001 |
| **Blood loss anemia** | 27912 (13%) | 17828 (14.2%) | 10084 (11.4%) | 0.349 |
| **Systemic** | | | | |
| **Sepsis** | 34557 (16.2%) | 19535 (15.6%) | 15022 (17%) | <0.001 |
| **Immunocompromised state (except HIV)** | 681 (0.3%) | 394 (0.3%) | 287 (0.3%) | 0.693 |
| **Sarcoidosis** | 506 (0.2%) | 249 (0.2%) | 257 (0.3%) | <0.001 |
| **Liver cirrhosis** | 9152 (4.3%) | 6028 (4.8%) | 3124 (3.5%) | <0.001 |
| **Amyloidosis** | 432 (0.2%) | 301 (0.2%) | 131 (0.1%) | <0.001 |
| **Orthostatic hypotension** | 2552 (1.2%) | 1727 (1.4%) | 825 (0.9%) | <0.001 |
| **Renal** | | | | |
| **Chronic kidney disease** | 71541 (33.4%) | 43484 (34.7%) | 28057 (31.7%) | <0.001 |
| **ESRD** | 17231 (8.1%) | 10649 (8.5%) | 6582 (7.4%) | <0.001 |
| **Dialysis dependence** | 11162 (5.2%) | 6960 (5.6%) | 4202 (4.7%) | <0.001 |
| **Acute Kidney Injury** | 80869 (37.8%) | 49114 (39.2%) | 31755 (35.9%) | <0.001 |
| **In-hospital hemodialysis** | 10584 (4.9%) | 6690 (5.3%) | 3894 (4.4%) | <0.001 |
| HIV: human immunodeficiency; ICD: Implantable cardioverter-defibrillator; CVA: cerebrovascular accident; NSTE: non-ST elevation; STEMI: ST elevation Myocardial Infarction; PCI: percutaneous coronary intervention; PRBC: packed red blood cell; CABG: coronary artery bypass grafting; AFib: Atrial fibrillation; TIA: transient ischemic attack; COPD: chronic obstructive pulmonary disease; UGI: Upper gastrointestinal; LGI: Lower gastrointestinal; IBD: inflammatory bowel disease; AV: atrioventricular; LBBB: left bundle branch block; OSA: obstructive sleep apnea; VTE: venous thromboembolism; SVT: supraventricular tachycardia; VT: ventricular tachycardia; PEG: Percutaneous endoscopic gastrostomy; ESRD: end stage renal disease; COVID-19: coronavirus disease 2019. | | | | |

**Supplementary Figure 2.** Sex-stratified trends in (A) mean length of hospital stay and (B) Inflation adjusted hospital cost among AFib patients with NSTEMI.


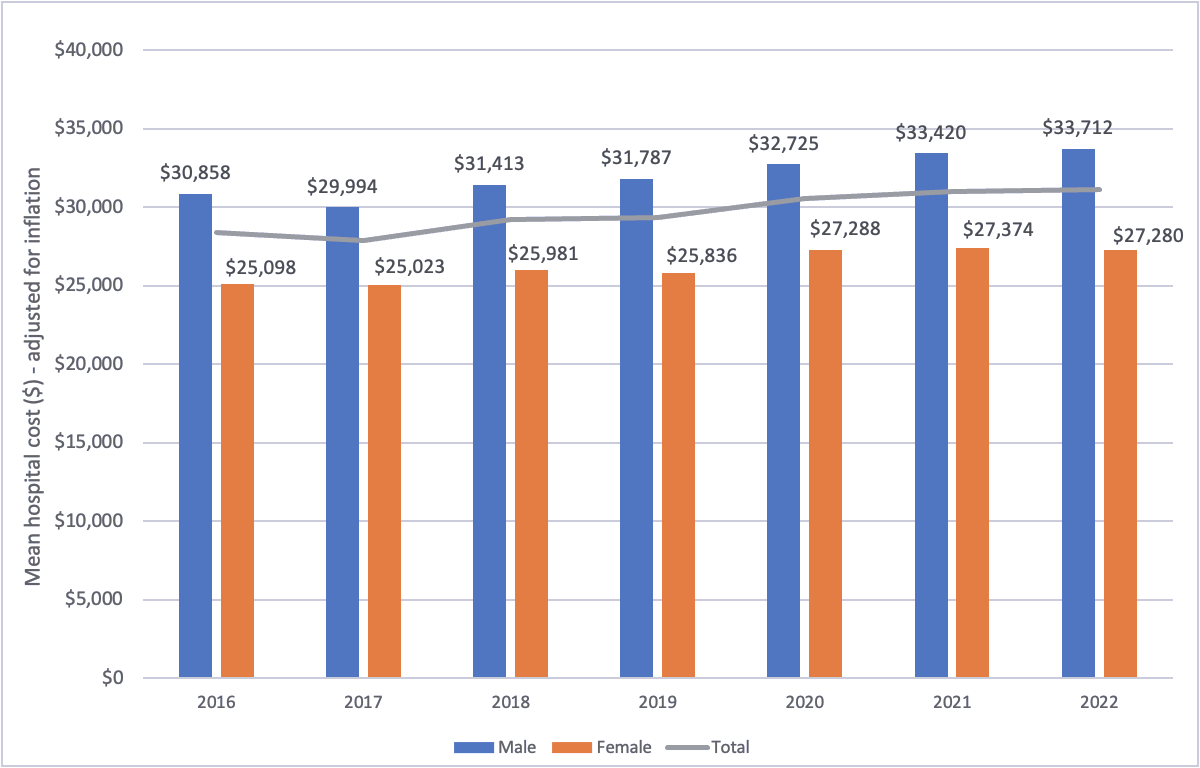


*P-trend for males: p<0.001; p-trend for females: p<0.001.

**
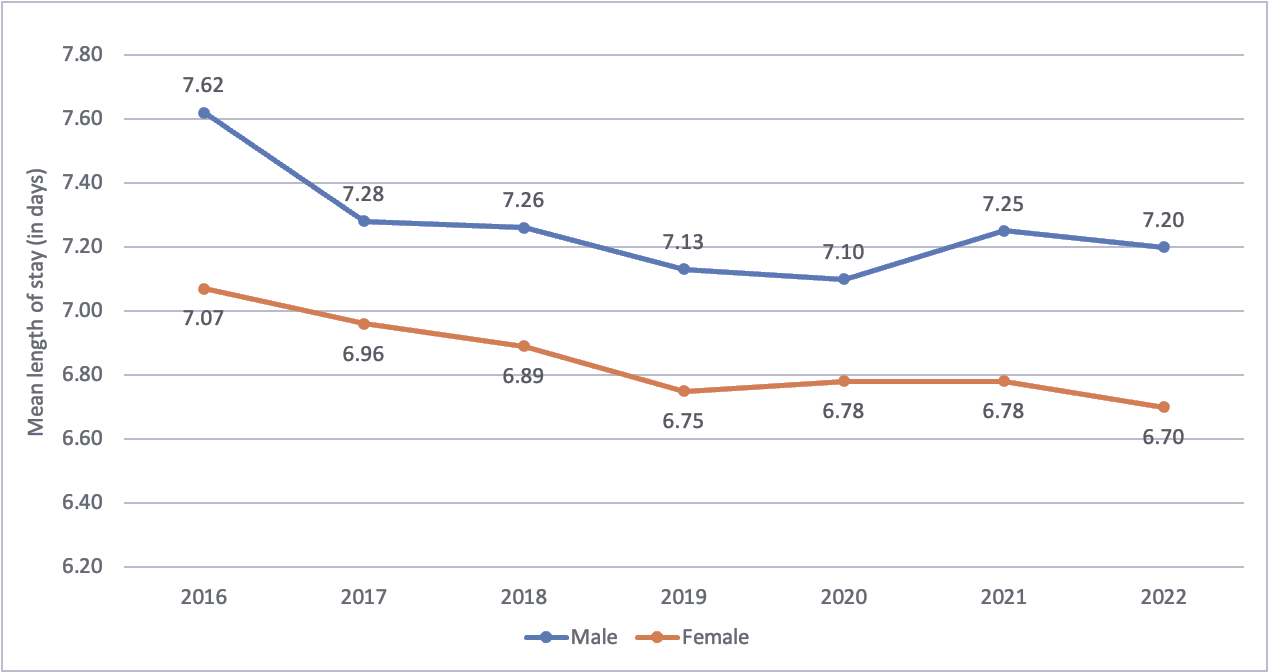
**

*P-trend for males: p<0.001; p-trend for females: p<0.001.

**Supplementary Figure 3.** Sex-stratified trends in revascularization strategies among AFib patients with NSTEMI.


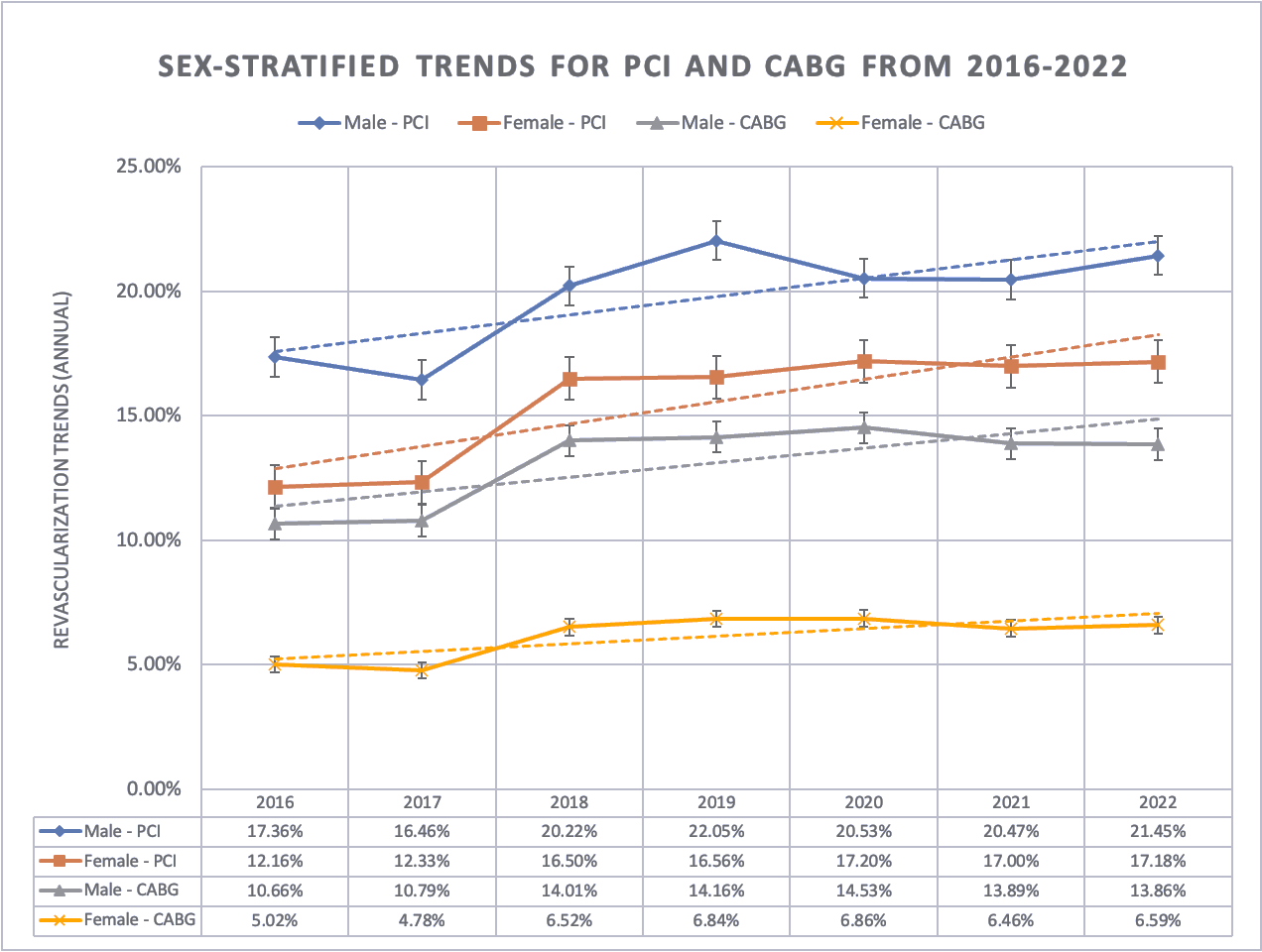


*P-trend for male-PCI: <0.001; P-trend for female-PCI: <0.001; P- for interaction (gender*PCI): <0.001

P-trend for male-CABG: <0.001; P- trend for female-CABG: <0.001; P-for interaction (gender*CABG): <0.001


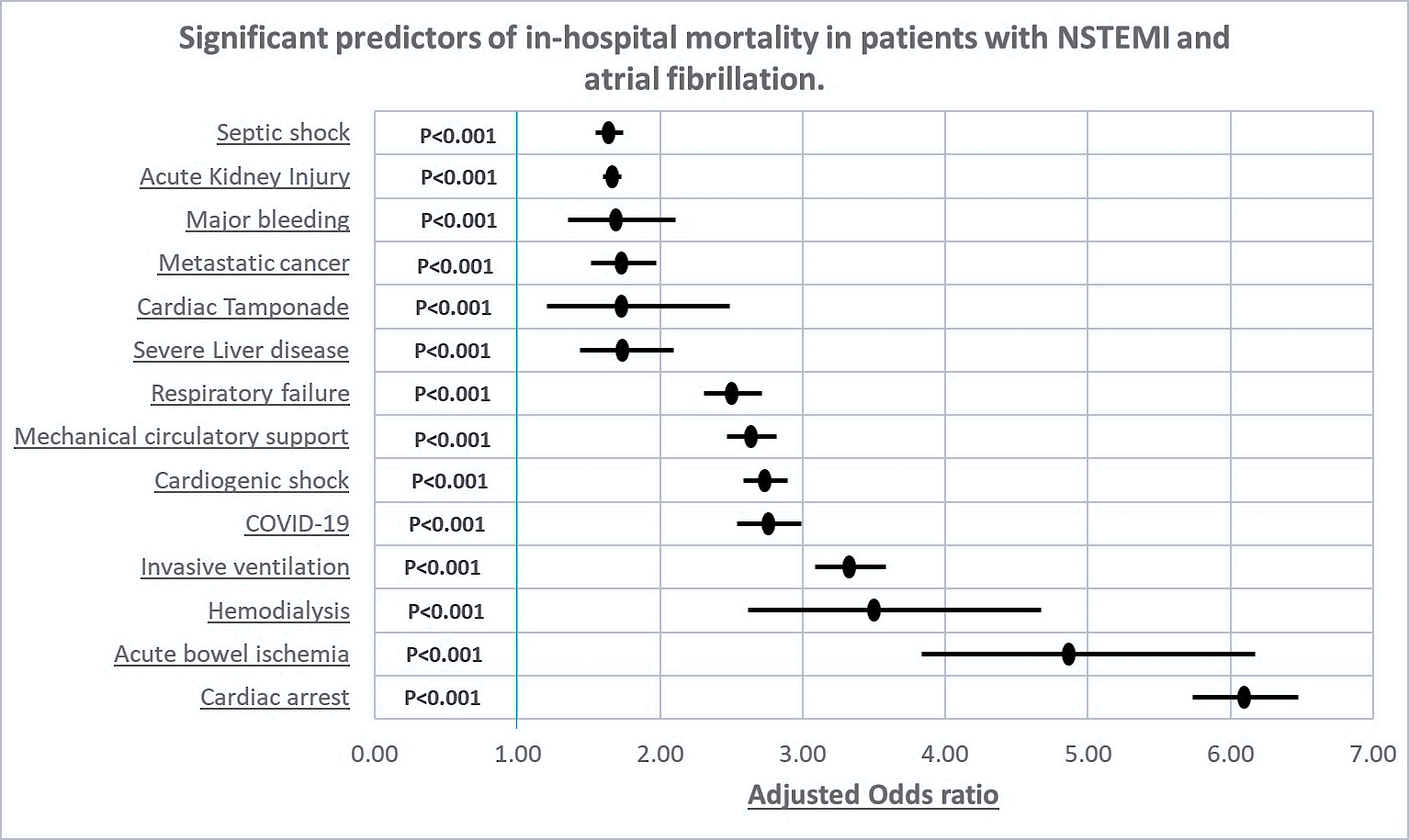
**Supplementary Figure 4.** Significant predictors of in-hospital mortality in AFib patients with NSTEMI.
